# Resveratrol Pretreatment Improved Heart Recovery Ability of Hyperglycemic Bone Marrow Stem Cells Transplantation in Diabetic Myocardial Infarction by Down-Regulating MicroRNA-34a

**DOI:** 10.3389/fphar.2021.632375

**Published:** 2021-04-20

**Authors:** Fengyun Zhang, Kun Wang, Fei Gao, Yongli Xuan, Xiaohong Liu, Zhuoqi Zhang

**Affiliations:** ^1^ The Affiliated Hospital of Xuzhou Medical University, Xuzhou, China; ^2^ Department of Cardiology, First People’s Hospital of Suqian, Suqian, China; ^3^ Department of Cardiology, Institute of Cardiovascular Research, Affiliated Hospital of Xuzhou Medical University, Xuzhou, China; ^4^ Department of Neurology, Affiliated Hospital of Xuzhou Medical University, Xuzhou, China; ^5^ Department of Cardiology, Affiliated Hospital of Xuzhou Medical University, Xuzhou, China

**Keywords:** resveratrol, microrna-34a, bone marrow-derived mesenchymal stem cells, diabetes mellitus, myocardial infarction, senescence

## Abstract

**AIM:** To examine the effect of resveratrol (RSV) on bone marrow mesenchymal stem cells (BMSCs) under hyperglycemic conditions and on BMSCs transplantation in diabetic rats with myocardial infarction (MI).

**METHODS:** *In vitro*, BMSCs were isolated from 3-week-old male Sprague Dawley (SD) rats and cultured under hyperglycemic conditions for up to 28 days. Cell viability was analyzed by cell counting kit-8 (CCK-8) assays. The expression of miR-34a was measured by RT-qPCR. Western blotting was used to examine the protein expression of SIRT1, P21, P16, VEGF and HIF-1α. A senescence-associated β-galactosidase assay was used to examine the senescence level of each group. *In vivo*, a diabetes model was established by feeding rats a high-sugar and high-fat diet for 8 weeks, injecting the animals with streptozotocin (STZ) and continuing high-sugar and high-fat feeding for 4 additional weeks. Then, left anterior descending coronary artery (LAD) cessation was used to established the myocardial infarction (MI) models. Each group of rats was transplanted with differentially preconditioned BMSCs after myocardial infarction. Ultrasound was used to analyze cardiac function 1 and 3 weeks after the operation, and frozen heart sections were used for immunohistochemical analysis, Masson staining and CD31 measurement. In addition, ELISA analysis of serum cytokine levels was performed.

**RESULTS:** This study showed that the viability of BMSCs cultured under hyperglycemic conditions was decreased, the cells became senescent. Besides, an obviously increased in the expression of miR-34a was detected. Moreover, RSV preconditioning reduced the expression of miR-34a in BMSCs after high glucose stimulation and rejuvenated BMSCs under hyperglycemic conditions. Further analysis showed that the transplantation of RSV-BMSCs were benefit to heart recovery following infarction in diabetic rats, promoted proangiogenic factor release and increased arteriole and capillary densities.

**CONCLUSION:** RSV rejuvenated BMSCs after chronic hyperglycemia-induced senescence by interacting with miR-34a and optimized the therapeutic effect of BMSCs on diabetes with myocardial infarction.

## Introduction

Cardiovascular diseases are the leading causes of morbidity and mortality worldwide and affect millions of individuals each year. Among which complicated with diabetes mellitus (DM) develop a two to five fold higher risk of heart failure compared with those without (GBD 2017 Causes of Death Collaborators, 2018). It is predicted that the prevalence of patients with DM will increase to 5.4% by the year 2025 and will affect about 300 million people worldwide (Kannel and McGee, 1979; King et al., 1998; Kanters et al., 1999). The huge health and economic burden promote us to take strategies to relieve the cardiac injury in DM patients.

In recent years, stem cell therapy has attracted much attention in promoting myocardial repair and the recovery of cardiac function after myocardial infarction, and considerable results in animal experiments and clinical trials have been achieved (Trounson and McDonald, 2015). Besides, stem cells infusion is reported to ameliorate cardiac fibrosis and dysfunction in diabetic cardiomyopathy rats (Jin et al., 2020), and promote angiogenesis, decrease the infiltration of immune cells and collagen deposition in diabetes (Ammar et al., 2015). However, diabetic stem cells are significantly impaired in their ability to improve cardiac function after myocardial infarction compared with control ones (Govaert et al., 2009). Studies have shown that bone marrow mesenchymal stem cells (BMSCs) cultured under high glucose conditions display a senescent phenotype (Chang et al., 2015), which may explain the low efficiency of autologous stem cell transplantation in diabetic patients with myocardial infarction. Therefore, improving the function and transplantation ability of diabetic BMSCs has become an appealing topic.

Resveratrol (RSV) is a nonflavonoid polyphenol compound that can inhibit formation of atherosclerotic plaques and reduce inflammation in the vascular endothelium. RSV has been shown to prevent the progression of heart failure and improve cardiac function and survival in animal models of myocardial infarction-induced heart failure. Studies have also shown that systemic RSV combined with the transplantation of RSV-preconditioned stem cells can maximize the antifibrotic effects in the treatment of diabetic cardiomyopathy (ShamsEldeen et al., 2019). However, whether RSV can rejuvenate BMSCs and improve transplantation ability in DM rats with myocardial infarction and the precise mechanism is not clear.

MicroRNAs are endogenous noncoding RNAs. Studies have shown that a variety of miRNAs, such as miR-25 (Wang et al., 2020) and miR-34a (Shao et al., 2018), play important roles in the occurrence and development of cardiovascular diseases. Among them, miR-34a plays an important role in promoting the aging process in BMSCs (Fulzele et al., 2019). Our previous study showed that the expression of miR-34a increased significantly upon ischemic/hypoxic condition, and miR-34a mimic pretreated BMSCs exhibited decreased cell ability, increased cell apoptosis rate and cell senescence under the ischemic and hypoxic environment (Zhang et al., 2015). Studies have shown that RSV protects cardiomyocytes against hypoxia/reoxygenation injury through the miR-34a/SIRT1 signaling pathway (Yang et al., 2016). Whether miR-34a is involved in the effects of RSV on BMSCs under hyperglycemic conditions needs further study.

Based on this background, the purpose of the present study was to examine whether RSV could attenuate BMSCs senescence under high glucose conditions, improve BMSCs transplantation ability in rats with diabetes and myocardial infarction and provide a strategy for the clinical application of autologous diabetic BMSCs transplantation in the treatment of myocardial infarction.

## Materials and methods

### Isolation and Culture of Bone Marrow Mesenchymal Stem Cells

BMSCs were cultured using the whole bone marrow adherence method, as described previously (Zhang et al., 2015). Briefly, total bone marrow was harvested from the femora of rats and plated in 21 cm^2^ culture flasks in Dulbecco’s modified Eagle’s medium (DMEM; Gibco; Thermo Fisher Scientific, Inc., Waltham, MA, United States) supplemented with 10% fetal bovine serum (Beyotime Institute of Biotechnology, Haimen, China) and 1% penicillin/streptomycin (Beyotime Institute of Biotechnology, Haimen, China) at 37°C with 5% CO_2_. After 3 days, the medium was replaced, and the nonadherent cells were discarded. The medium was completely replaced every 3 days thereafter. Approximately 7–9 days after seeding, the cells became 70–80% confluent. The adherent cells were released from the dishes using 0.25% trypsin (Beyotime Institute of Biotechnology, Haimen, China) and expanded at a 1:2 or 1:3 dilution. All in this study were performed using bone marrow mesenchymal stem cells from the third to fifth generation.

### Resveratrol Treatment

RSV was purchased from Sigma. RSV was dissolved in dimethyl sulfoxide (DMSO) at a concentration of 1 M and then diluted with media to 1 mM (containing 0.1% DMSO). BMSCs were seeded at a density of 2 × 10^4^ cells/cm^2^ and treated with 0, 1, 2, and 3 μM RSV. After 96 h, BMSCs viability was analyzed.

### Cell Viability Assay

BMSCs viability was examined using the cell counting kit-8 (CCK-8) assay (Dojindo, Japan) in accordance with the manufacturer’s protocols. Cells were seeded in a 96-well plate (5,000 cells per well), and then 10 μl of CCK-8 was added to the culture medium and incubated for 2 h. The absorbance of each well was measured at 450 nm. All data were calculated from triplicate samples.

### Establishment of the High Glucose Bone Marrow Mesenchymal Stem Cells Model

BMSCs were cultured in normal glucose (5.5 mM glucose) or DMEM/HG (25 mM glucose). The cells were passaged when they had grown to 70∼80% confluence and were then cultured for 4 weeks for follow-up experiments. The HG group was then treated with different stimuli including RSV, miR-34a mimic or inhibitor.

### Cell Transfection

BMSCs from each group were seeded in a 6-well plate at 2 × 10^5^ cells per well and cultured overnight. BMSCs were transfected for 48 h with different concentrations (20, 30, 40, and 50 nM) of the miR-34a mimic/inhibitor to overexpress/silence miR-34a, respectively, according to the instructions of X-treme siRNA transfection reagent. Real-time fluorescent quantitative polymerase chain reaction (RT-qPCR) was used to determine the transfection efficiency.

### Extraction and Quantitative RT-Polymerase Chain Reaction

After the BMSCs in each group were treated, total RNA was extracted by the TRIzol method, and RT-qPCR was carried out using a two-step method according to the instructions. The upstream and downstream primers of miR-34a and U6 were designed and synthesized by Beijing Tiangen Biochemical Technology Co., Ltd. The 2-^ΔΔCt^ method was used for relative quantitative analysis. Table 1 presents all related gene sequences.

**TABLE 1:** Primers for qRT-PCR and oligonucleotide.

Name		Sequence
**qPCR**		
miR-34a		5'-UGG​CAG​UGU​CUU​AGC​UGG​UUG​UU-3'
U6	F	5'-CCT​GCT​TCG​GCA​GCA​CA-3'
	R	5'-AAC​GCT​TCA​CGA​ATT​TGC​GT-3'
**Oligonucleotide**		
miR-34a mimic		UGG​CAG​UGU​CUU​AGC​UGG​UUG​UU CAA​CCA​GCU​AAG​ACA​CUG​CCA​UU
Negative Control (NC) mimic		UUC​UCC​GAA​CGU​GUC​ACG​UTT ACG​UGA​CAC​GUU​CGG​AGA​ATT
miR-34a inhibitor		UGG​CAG​UGU​CUU​AGC​UGG​UUG​UU
NC inhibitor		CAG​UAC​UUU​UGU​GUA​GUA​CAA

### Western Blot Analysis

Total cellular protein was extracted from BMSCs or heart sections from the different treatment groups. The cells or tissues were washed in cold PBS, and total protein was extracted using RIPA lysis buffer supplemented with a protease inhibitor cocktail. The total protein concentration was analyzed using the bicinchoninic acid assay according to the manufacturer’s instructions. Total extracts (50 μg of total protein) were separated by 8 and 10% SDS-PAGE gels and transferred onto polyvinylidene difluoride (PVDF) membranes. After the membranes were blocked with fat-free milk, the membranes were incubated overnight at 4°C with the following primary antibodies: p21 (1:1,000), p16 (308) (1:1,000) (both from Hua’an Biotechnology Co., Ltd., Zhejiang, China), SIRT1 (1:1,000) (Cell Signaling Technology), HIF-1α (1:1,000) (Santa Cruz Biotechnology Co., Ltd., United States) and VEGF (1:1,000) (Servicebio, Wuhan, China). The membranes were washed with TBST and then incubated with secondary antibodies (goat anti-rabbit IgG) diluted at 1:5,000 for 1 h at room temperature. ImageJ was used to quantify the band intensities after the membranes were stained according to the BeyoECL Plus kit instructions.

### Senescence-Associated β-galactosidase Staining

SA-β-gal staining was performed using a senescence-associated β-galactosidase staining kit (Beyotime Biotechnology, China) according to the manufacturer's protocol. The cells were fixed in β-galactosidase fixation buffer for 15 min at room temperature and were then stained with SA-β-gal staining solution overnight at 37°C. SA-β-gal-positive cells exhibited blue coloration. The number of positive cells was counted under a phase-contrast microscope. The experiment was repeated three times in each group.

### Establishment of an Sprague Dawley Rat Model of Diabetes With Myocardial Infarction

After 1 week of adaptive feeding, normal male SD rats were fed a high-sugar and high-fat diet for 8 weeks. After 12 h of fasting without water, a single intraperitoneal injection of 1% STZ in citrate buffer (25 mg/kg) was administered to establish a type 2 diabetes model. At 72 h after the injection and after being fasted without water for 8 h, blood samples from the tail vein of each rat was used to measure the blood glucose level; a blood glucose level ≥ 11.1 mmol/L was considered successful. After successful establishment of the type 2 diabetes model, the rats continued to be fed a high-sugar and high-fat diet for 4 weeks for subsequent experiments. To establish the MI model, the rats in each group were fasted for 8 h and were not allowed water for 4 h before the operation. After successful anesthesia, the rat was connected to a BL-420S biorecorder and ventilator, and the parameters of the ventilator were adjusted as follows: 30 ml/kg tidal volume, 80 times/min respiratory rate, and 1:2 respiratory ratio. After sterilization and thoracotomy, a 6–0 nylon thread was used to ligate the anterior descending coronary artery at the upper 1/3 of the connection between the edge of the left atrial appendage and the cardiac apex. After treatments in each group, the serum-free BMSCs suspension was injected intramuscularly at four sites around the border zone of the infarcted heart.

### Echocardiography

Rats in each experimental group underwent echocardiography (Philips IE33; probe: Philips S12–4) at 1 and 3 weeks after surgery. The long axis view of the left ventricle was taken during the ultrasound examination, and the detection indicators were measured in three consecutive cardiac cycles and averaged.

### Masson’s Staining

After echocardiography assessment at 3 weeks after MI, all rats were sacrificed, and the heart tissues were harvested, embedded, and sectioned. The infarction size of the rat heart, as evidenced by fibrosis, was examined by a Masson’s staining kit according to the manufacturer's protocol (HT15, Sigma).

### Immunohistochemistry

To determine the blood vessel density in the heart tissue in the different groups, the heart sections were immunohistochemically stained for CD31 (1:2,000; Servicebio, Wuhan, China). The capillary and arteriole densities are expressed as the average number of CD31-positive blood vessels per field (×10).

### Cytokine Measurement Via Enzyme-Linked Immunosorbent Assay

VEGF and HIF-1α concentrations were assessed in the serum of rats by standard sandwich ELISA (Wuhan Yunkelong Technology Co., Ltd., Hubei, China) according to the manufacturer’s instructions. The concentrations of VEGF and bFGF are expressed in nanograms per milliliter and were calculated based on calibration curves constructed from serial dilutions of recombinant standards. The sensitivity of the VEGF and bFGF assays was 2 pg/ml.

### Statistical Analysis

All data were analyzed using GraphPad Prism seven and are expressed as the mean ± standard deviation (SD). Comparisons between two groups were performed using Student’s t tests, while the significance of differences between three or more experimental groups was determined by one-way analysis of variance. A value of *p* < 0.05 was considered statistically significant.

## Results

### Hyperglycemia Damages Bone Marrow Mesenchymal Stem Cells Functions

**FIGURE 1:**
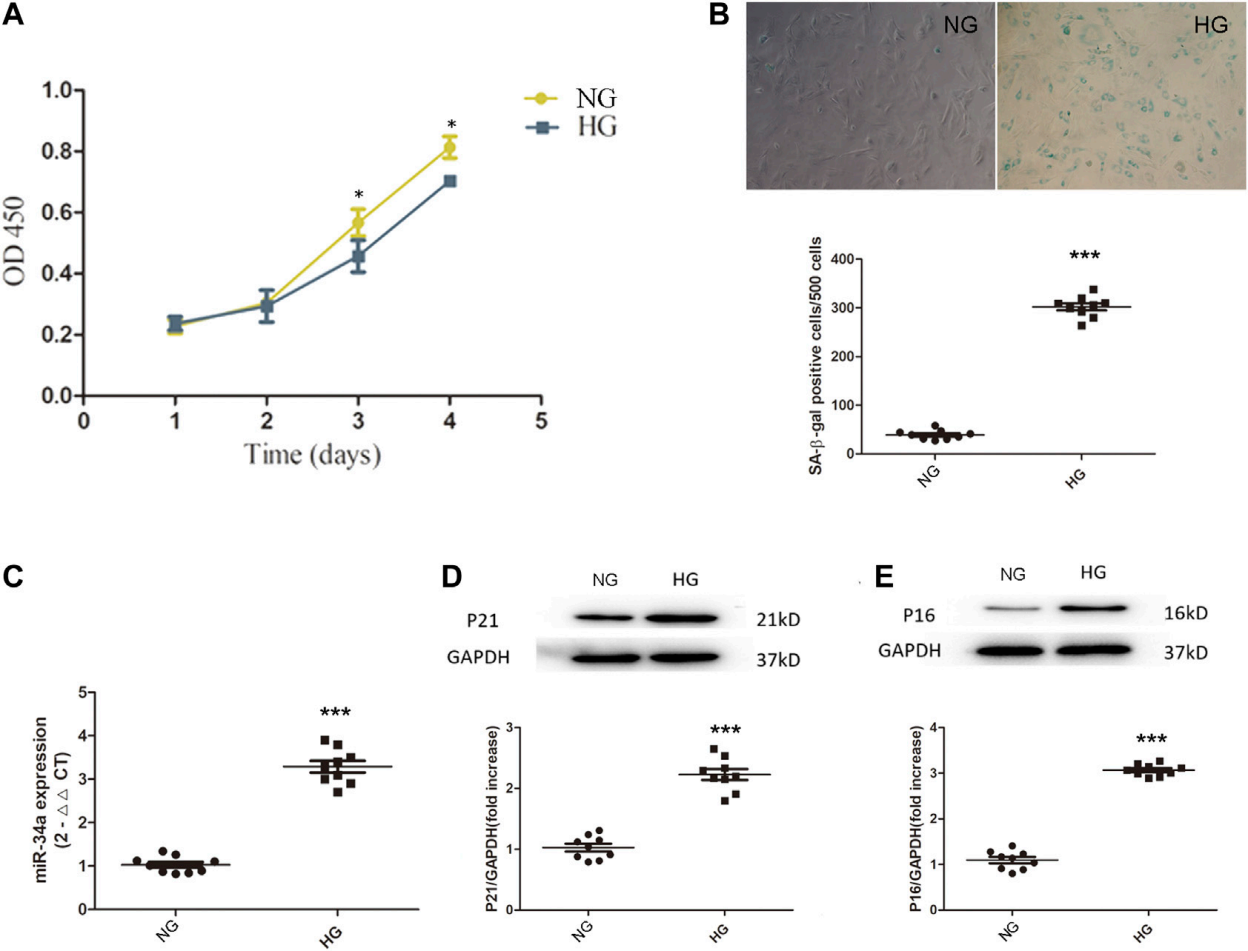
Hyperglycemia damage BMSCs’ cell function. **(A)** BMSCs were cultured in normal-glucose medium and high-glucose medium. The viability of BMSCs was detected by CCK8 assay. ^*^ *p* < 0.05. **(B)** SA-β-galactosidase staining experiment was used to detect the senescence level between NG group and HG group cells. ***p < 0.001. **(C)** the expression of miR-34a was determined by qRT-PCR. ***p < 0.001 **(D,E)** Western blot analysis showed the expression levels of senescence-related proteins P21 and P16 in NG group and HG group. ***p < 0.001.

The CCK-8 results showed that under hyperglycemic conditions, BMSCs exhibited impaired cell proliferation (Figure 1A). SA-β-galactosidase staining showed that BMSCs exposed to high glucose conditions became senescent over time (Figure 1B). In addition, the expression of p21 and p16 in BMSCs cultured in high glucose was also significantly higher than that in BMSCs cultured in normal glucose (Figures 1D,E), which indicated that under chronic hyperglycemic conditions, BMSCs had reduced viability and a significant tendency to become senescent. We then used qRT-PCR to measure the expression of miR-34a and found that miR-34a expression was significantly increased in BMSCs under high glucose conditions (Figure 1C), suggesting a connection between miR-34a and cellular dysfunction under hyperglycemic conditions.

### Resveratrol Treatment Increases Bone Marrow Mesenchymal Stem Cells Viability and Reduces miR-34a Expression Under Hyperglycemic Conditions

**FIGURE 2:**
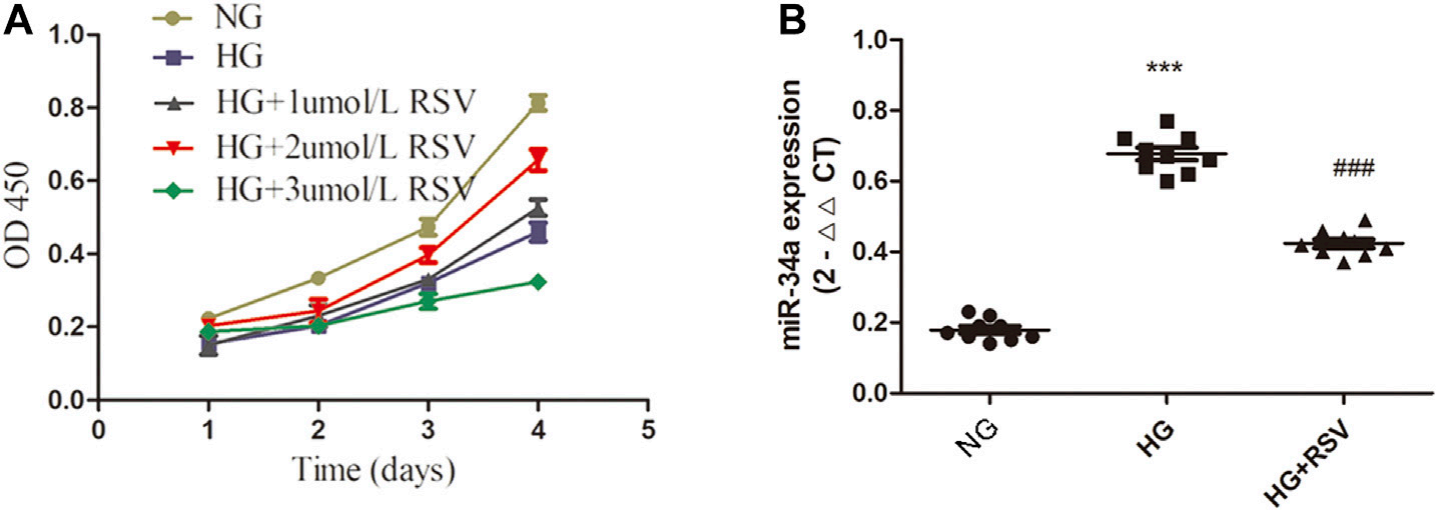
RSV treatment increases BMSCs viability and reduces miR-34a expression under hyperglycemic conditions. **(A)** The effects of different concentrations (1umol/L, 2umol/L, 3umol/L) of RSV pretreatment for different time on the activity of BMSCs. **(B)** qRT-PCR analysis was applied to detect the expression of miR-34a in each group. ***p < 0.001 vs NG, ^###^ *p* < 0.001 vs HG.

RSV has been reported to have antioxidative, anti-inflammatory, antiapoptotic and other effects (Hu and Li, 2019; Yanez et al., 2019; Zhang X. L. et al., 2020). To clarify the effect of RSV on stem cells, BMSCs were cultured with different concentrations of RSV for different times. As shown in Figure 2A, RSV promoted the viability of BMSCs at a concentration of 2 µM and a treatment time of 96 h. To further confirm the interaction of RSV and miR-34a in BMSCs under high glucose conditions, we used qRT-PCR to measure the expression of miR-34a (Figure 2B), and the results showed that hyperglycemia increased miR-34a expression, while RSV reversed this effect under hyperglycemic conditions.

### Resveratrol Treatment Reduces Bone Marrow Mesenchymal Stem Cells Senescence Under Hyperglycemic Conditions by Regulating miR-34a Expression

**FIGURE 3:**
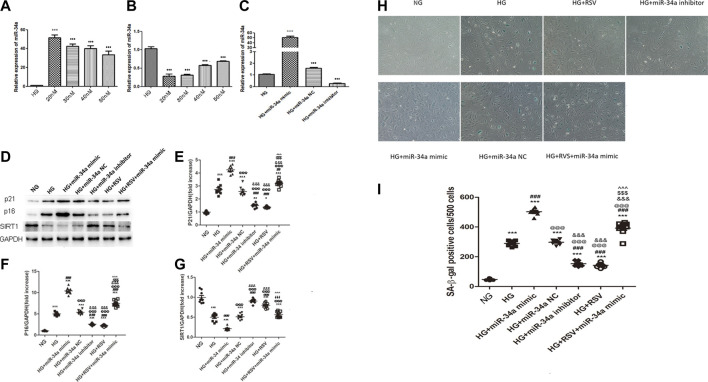
RSV treatment reduces BMSCs senescence under hyperglycemic conditions by regulating miR-34a expression **(A–C)** Quantitative RT-PCR was used to determine the optimal transfection concentration of miR-34a mimic and inhibitor of different concentrations. ***p < 0.001 vs HG. **(D–G)** Western blot analysis shows the expression of SIRT1and senescence-related proteins P21 and P16 in each. group of cells. Each column represents mean ± SD from three independent experiments. **p < 0.01, ***p < 0.001 vs NG. ^###^ *p* < 0.001 vs HG. ^@@@^ *p* < 0.001 vs HG + miR-34a mimic. ^&&&^ *p* < 0.001 vs HG + miR-34a NC. ^$$$^ *p* < 0.001 vs HG + miR-34a inhibitor. ^^^*p* < 0.001 vs HG + RSV. **(H,I)** SA-β-galactosidase staining is used to judge the senescence degree of each group of cells. ***p < 0.01, ***p < 0.001 vs NG. ^###^ *p* < 0.001 vs HG. ^@@@^ *p* < 0.001 vs HG + miR-34a mimic. ^&&&^ *p* < 0.001 vs HG + miR-34a NC. ^$$$^ *p* < 0.001 vs HG + miR-34a inhibitor. ^^^*p* < 0.001 vs HG + RSV.

To examine whether RSV participates in the regulation of BMSCs function under high glucose conditions through regulating miR-34a, we transfected miR-34a mimic, miR-34a inhibitor or miR-34a NC into BMSCs. To verify the optimal transfection efficiency of the miR-34a mimic/inhibitor, we transfected BMSCs with different concentrations of the miR-34a mimic and inhibitor, and the results showed that the optimal transfection concentration was 20 nM (Figures 3A–C). Western blotting was used to detected the expression of P21 and P16 in each group. As shown in Figures 3D,E, P21 protein expression increased in the HG group compared with the NG group, miR-34a mimic aggravated the P21 expression of HG, while RSV and miR-34a inhibitor reversed this effect of HG. Furthermore, compared with HG + miR-34a mimic group, RSV alleviated the effect of the miR-34a mimic on aggravating P21 protein expression. Similar result could be found in p16 protein expression (Figures 3D–F).

SIRT1 was verified to be the direct target of miR-34a in our previous study, and we further examined whether SIRT1 could be regulated by RSV upon hyperglycemia. As shown in Figure 3D, HG reduced the expression of SIRT1, and miR-34a mimic aggravated the down-regulation of SIRT1 expression, while the miR-34a inhibitor increased the SIRT1 expression. Besides, RSV also reversed this effect of HG on SIRT1 expression and RSV could alleviated the effect of the miR-34a mimic on SIRT1 expression.

To further examine the senescence of cells in each group, we performed β-galactosidase staining to determine the effects of RSV and miR-34a on the regulation of BMSCs senescence. Consistent with the expression of senescence-related proteins, we found that RSV alleviated BMSCs senescence by inhibiting the hyperglycemia-induced expression of miR-34a (Figures 2H,I).

### Resveratrol-Bone Marrow Mesenchymal Stem Cells Transplantation Improves Cardiac Function Following Infarction in Diabetic Rats

**FIGURE 4:**
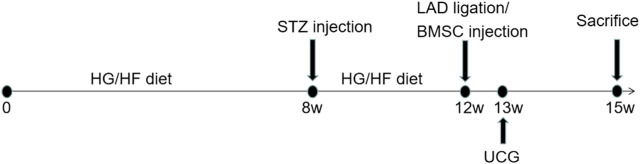
Experimental protocol of the *in vivo* test. Normal male SD rats were fed with high-sugar and high-fat diet for 8 weeks. 1% STZ was injected with 25 mg/kg establish a type 2 diabetes model. The rats continued to be fed with high-sugar and high-fat diet for 4 weeks. Then the LAD were ligated and BMSCs with different stimulation were transplanated into the border of the infarction area. 1 week after this surgery, echocardiography was taken to measured the cardiac function and 3 weeks after, these rat were sacrificed for further studies.

**FIGURE 5:**
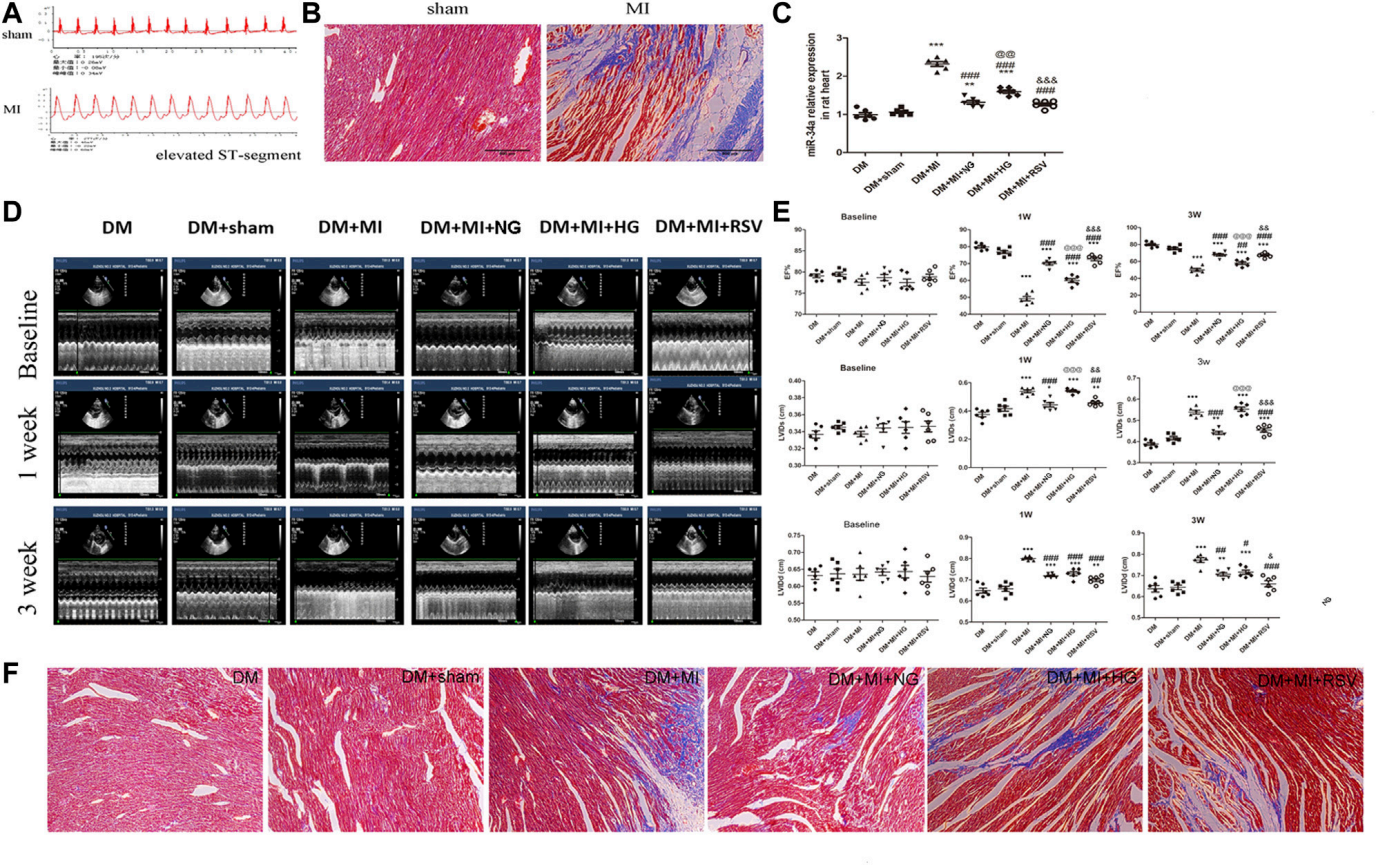
RSV-BMSC transplantation improves cardiac function following infarction in diabetic rats **(A,B)** Electrocardiogram and Masson staining were used to detect the establishment of a rat model of diabetes with myocardial infarction. **(C)** qRT-PCR analysis was applied to detect the expression of miR-34a in each group. **(D,E)** Echocardiography showed the heart function in the control group and the experimental group at 1 and 3 weeks after operation. **(F)** Masson staining were used to detect in the control group and the experimental group at 3 weeks after operation. ***p < 0.001 vs DM. ^##^ *p* < 0.01, ^###^ *p* < 0.001 vs DM + MI. ^@@^ *p* < 0.01, ^@@@^ *p* < 0.001 vs DM + MI + NG. ^&&^ *p* < 0.01, ^&&&^ *p* < 0.001 vs DM + MI + HG.

*In vivo* experiments were performed following the protocol as shown in Figure 4. To further verify the therapeutic effect after transplantation, we injected BMSCs from each group immediately after myocardial infarction. The ST segment of the electrocardiogram was significantly elevated after the anterior descending branch was ligated, which is an indicator of myocardial infarction (Figure 5A). Ultrasonography was performed to observe changes in heart function in rats in each group at 1 and 3 weeks. Then, the rats were sacrificed, and the frozen heart sections was analyzed. Masson staining showed that the myocardial infarction group had substantial blue-stained fibrous tissue in the heart compared with that in the sham group (Figure 5B). QRT-PCR was performed to detect the expression of miR-34a in the MI hearts after different treatments (Figure 5C). MiR-34a was significant elevated in the MI group.

Compared with that in the sham group, the left ventricular ejection fraction (LVEF) in the MI group decreased at 1 and 3 weeks (Figures 5D,E), which suggested the myocardial contractility reduction in myocardial infarction. When BMSCs were transplanted into the infarction area, the LVEF was moderately elevated. However, compared with that in the NG BMSCs group, the LVEF was reduced in the HG BMSCs group, but this effect was partially ameliorated in the RSV HG group, indicating that RSV + HG BMSCs were superior to HG BMSCs in improving heart function following MI (Figures 5D,E). In addition, compared to those in the HG BMSCs group, the LV end-diastolic dimension (LVIDd) and end-systolic dimension (LVIDs) were significantly decreased in the RSV BMSCs group (Figures 5D,E). Furthermore, masson's trichrome staining was performed to detect the infarct size, and we could found that the infarct size was higher in the HG BMSCs group than in the NG BMSCs group (Figure 5F), while RSV BMSCs showed benefit to the cardiac recovery after myocardial infarction compared with those in the HG BMSCs group.

### Resveratrol-Bone Marrow Mesenchymal Stem Cells Enhance Angiogenesis in the Infarcted Hearts of Diabetes Mellitus Rats

**FIGURE 6:**
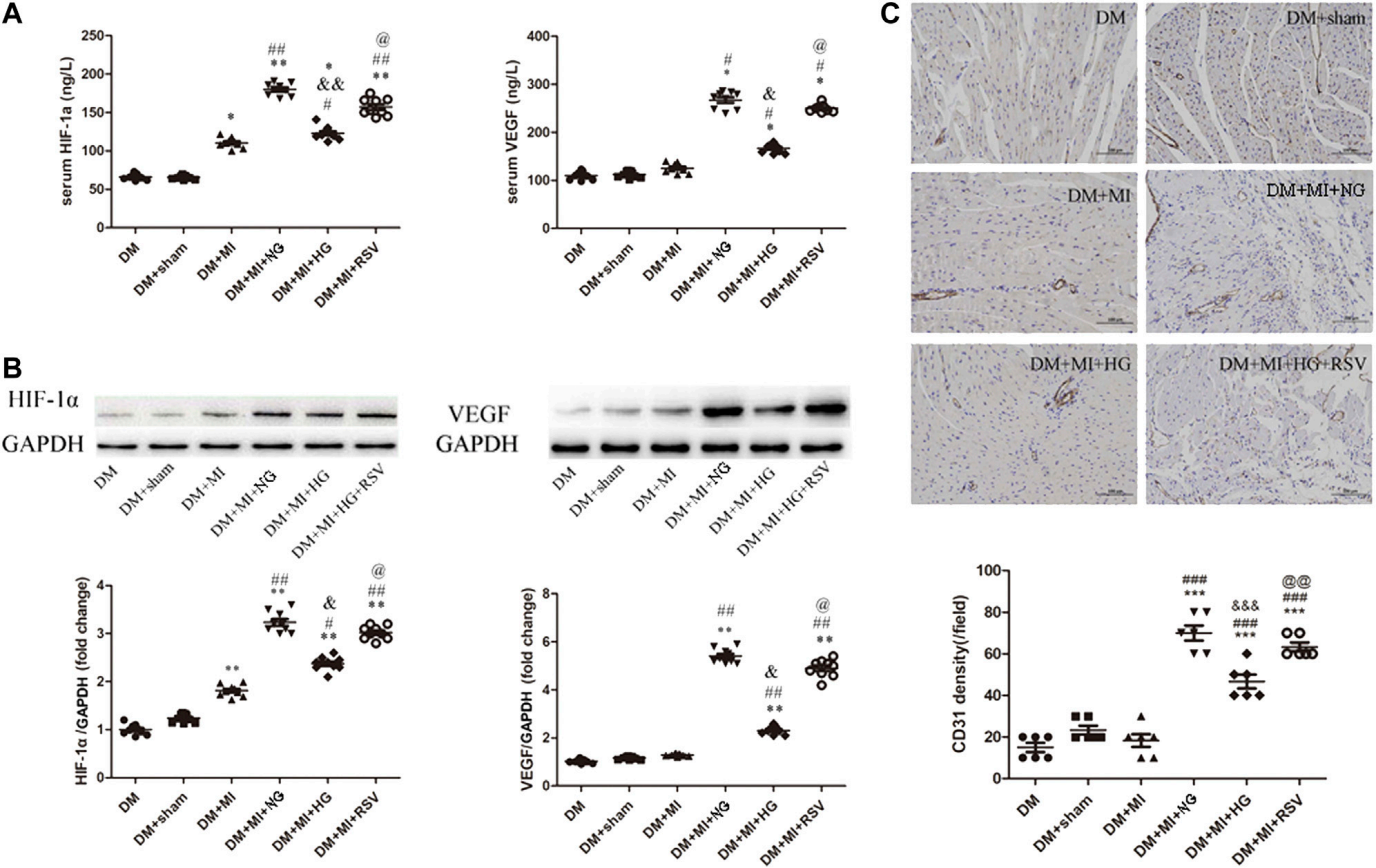
RSV-MSCs enhance angiogenesis in the infarcted hearts of DM rats **(A)** ELISA was used to detect the pro-angiogenic factor of HIF-1α and VEGF expression, **(B)** and WB was used to detect the protein expression of HIF-1α and VEGF in the heart section. **(C)** Representative images of CD31 staining and quantitative analysis of the density of arterioles of heart tissue from control group and experimental group. Scale bar = 100 μm. Data are expressed as the mean ± SEM. *p < 0.05 vs DM sham. ^##^ *p* < 0.01 vs DM + MI. ^&^ *p* < 0.001 vs DM + MI + NG. ^@^ *p* < 0.05 vs DM + MI + HG.

To determine the angiogenic effects of BMSCs transplantation, serum and heart sections were analyzed by ELISA and WB to determine the expression of the proangiogenic factors HIF-1α and VEGF, and the arteriole and capillary densities were examined by CD31 staining in rat hearts at 3 weeks after transplantation. As shown in Figure 6A, the expression of HIF-1α and VEGF was increased in the MI group compared with the sham group. In the NG BMSCs-treated group, the expression of these proteins was obviously increased compared with that in the MI group, but in the HG BMSCs transplant group, there was little change in HIF-1α or VEGF production. When RSV-preconditioned BMSCs were transplanted into the infarction area, proangiogenic factors were greatly increased compared with those in the HG BMSCs group. Consistent with these findings, similar results were found regarding the protein expression of HIF-1α and VEGF in frozen heart sections (Figure 6B). Compared with that in the MI group, the arteriole density was significantly increased in the NG BMSCs-treated group, but in the HG BMSCs-treated groups, there were only slight increases in CD31 expression and arteriole density (Figure 6C). Compared with the HG BMSCs-treated group, the RSV + HG BMSCs-treated group showed higher capillary density.

Collectively, these findings suggest that RSV-BMSCs transplantation enhances angiogenesis in infarcted hearts in DM rats, suggesting an effective method of autologous stem cell transplantation to treat diabetic patients with myocardial infarction.

## Discussion

Epidemiology, the mortality rate for cardiovascular diseases (CAD) is about 12% of total death causes and affected the population aged between 35 and 74 years (Moran et al., 2014). Diabetes mellitus (DM) increases the risk of developing cardiovascular disease. The prevalence of DM is estimated to increase to 5.4% in 2025 and will affect about 300 million population worldwide (Kannel and McGee, 1979; King et al., 1998; Kanters et al., 1999). Indeed, patients with type 2 diabetes who have not had a MI have a risk of infarction similar to that among nondiabetic patients who have had a prior MI (Haffner et al., 1998). Cardiovascular outcomes, hospitalization and prognosis are worse for patients with diabetes relative to those without. Therefore, strategies to help reduce the global burden of MI with DM are pivotal.

With the development of cell and tissue engineering, stem cells have rapidly become a hot topic in various fields due to their powerful reparative functions. In recent years, a large number of clinical and animal experiments have proven BMSCs transplantation can help reduce myocardial infarction injury and improve cardiac function (Liang et al., 2014; Li et al., 2018). MSCs transplantation showed a significant reduction of systolic blood pressure associated with improvement of cardiac contractility in the diabetes rats (Abdel Aziz et al., 2008). Among the different sources of MSCs, syngeneic BMSCs transplantation are better than allogeneic BMSCs transplantation on the long-term effects of recovery heart function resulting from the more cell survival due to low immunorejection (Mahmoud et al., 2019). However, in a study by Govaert et al. (2009), the researchers transplanted diabetic and normal mouse bone marrow mononuclear cells into the myocardial infarction area of diabetic mice and found that the transplanted cells from diabetic mice did not improve the ejection fraction in mice with myocardial infarction.

In-depth studies have shown that under high glucose conditions, BMSCs exhibit increased senescence and decreased proliferation. This may partly be regulated by regulating the Akt/mTOR pathway (Zhang et al., 2017). Senescent cells are major contributors to impaired function and increased mortality following MI, while pharmacological clearance of senescent cells improves survival and recovery in aged mice following acute myocardial infarction (Walaszczyk et al., 2019). In accordance with the previous study, the present study showed that under chronic hyperglycemic conditions, BMSCs showed impaired viability and became senescence, which can be seen in decreased CCK-8 vitality and inhibited expression of p21 and p16, as well as the SA-β-gal staining.

RSV is a nonflavonoid polyphenol compound. Research on the protective effect of RSV on the cardiovascular system has attracted much attention, and its antioxidative and antiatherosclerotic effects have been confirmed. ShamsEldeen et al. (2019) showed that systemic RSV combined with RSV-preconditioned mesenchymal stem cells to treat diabetic cardiomyopathy can maximize antifibrotic effects. In addition, studies have shown that RSV can reduce the senescence of adipose-derived mesenchymal stem cells (ADMSCs) and improve their paracrine functions, and this effect occurs by upregulating the expression of Pim-1 through the PI3K/AKT pathway (Lei et al., 2016). Whether RSV can rejuvenate BMSCs and improve transplantation ability in DM rats with myocardial infarction and the precise mechanism is not clear. In the present study, we found that RSV increased cell viability. Moreover, RSV could reverse the up-regulation of miR-34a mimic induced by hyperglycemia.

MiR-34a belongs to one of several evolutionarily conserved families of miRNAs, namely miR-34 (He et al., 2007). MiR-34a was expressed in almost every tissue but was scarcely expressed in lung tissue (Bommer et al., 2007). MiR-34a expression levels were significantly increased in the animal model of acute MI and in the aged hearts (Boon et al., 2013) and were strongly connected with heart remolding at 1 year after acute MI (Matsumoto et al., 2013). Furthermore, miR-34a-5p inhibition protected cardiomyocytes against hypoxia-induced cell injury (Shi et al., 2019). Results suggest that miR-34a can attenuate myocardial fibrosis in dilated cardiomyopathy by reducing type I collagen production, cell viability, and migration and increasing the apoptosis (Zhang Y. et al., 2020). In this study, we first showed that under high glucose conditions, the expression of miR-34a in BMSCs increased significantly. MiR-34a aggravated the hyperglycemia induced senescence, while RSV reversed the effect of the miR-34a mimic, as verified by decreased P21 and P16 protein expression and SA-β-gal staining. SIRT1 verified as miR-34a target gene in our previous study and could be negative regulated by miR-34a in MSCs apoptosis and senescence upon hypoxia and serum deprivation (H/SD) situation. In the present study, we found RSV could reverse the down-regulation of SIRT1 by hyperglycemia.

*In vivo*, we transplanted differentially stimulated BMSCs into the ischemic border. We found that in DM rats, the LVEF decreased significantly after LAD ligation. MSCs transplantion was reported to significantly elevate LVEF after MI both in clinical and animal studies (Fu et al., 2017; Kim et al., 2018). After BMSCs transplantation, the LVEF increased in the BMSCs group at both 1 week and 3 weeks and the infacrtion area reduced; additionally, RSV-treated BMSCs obviously increased the LVEF and decreased the myocardial fibrosis. RSV could protect BMSCs from hyperglycaemic injury and provide a promising role for autologous transplantion of diabetic BMSCs in treating myocardial infartion patients.

Angiogenesis, which is the formation of new blood vessels, is responsible for a wide variety of physio/pathological processes (Braile et al., 2020; Lin et al., 2020). In the present study, we found that the expression of VEGF and HIF-1α decreased in the serum and frozen tissue of DM MI rats, but after NG BMSCs transplantation into the heart, the expression of these proangiogenic factors increased, and RSV promoted the expression of proangiogenic factors and new blood vessel formation. These results suggested that RSV increased BMSCs transplantation ability under hyperglycemic conditions and promoted heart healing after MI.

Several limitations exist in our studies. First, we did not calculate the cell retention after RSV pre-treated BMSCs delivered into the infarcted myocardium, which is also very important for the researcher to detect the BMSCs transplantation ability into MI. Second, more precise underlying mechanism need to be investigated. Third, indicators of heart remodeling like collagen need to be tested to make this study more convincing.

In conclusion, we found that hyperglycemia impaired cell viability and induced cell senescence; during this process, the expression of miR-34a increased. Under hyperglycemic conditions, RSV-preconditioned BMSCs showed decreased expression of cellular senescence-related proteins, a reduction in the senescent phenotype and decreased miR-34a expression. *In vivo* experiments also confirmed that RSV-preconditioned BMSCs have superior transplantation ability in myocardial infarction and promote microangiogenesis and heart healing after myocardial infarction in DM rats.

## Data Availability

The raw data supporting the conclusions of this article will be made available by the authors, without undue reservation, to any qualified researcher.
