# Supplementary figures and images for: Resveratrol Pretreatment Improved Heart Recovery Ability of Hyperglycemic Bone Marrow Stem Cells Transplantation in Diabetic Myocardial Infarction by Down-Regulating MicroRNA-34a
Source: Front Pharmacol. 2021 Apr 20;12:632375. doi: 10.3389/fphar.2021.632375 (PMC8223511; doi:10.3389/fphar.2021.632375)

## Slide 1
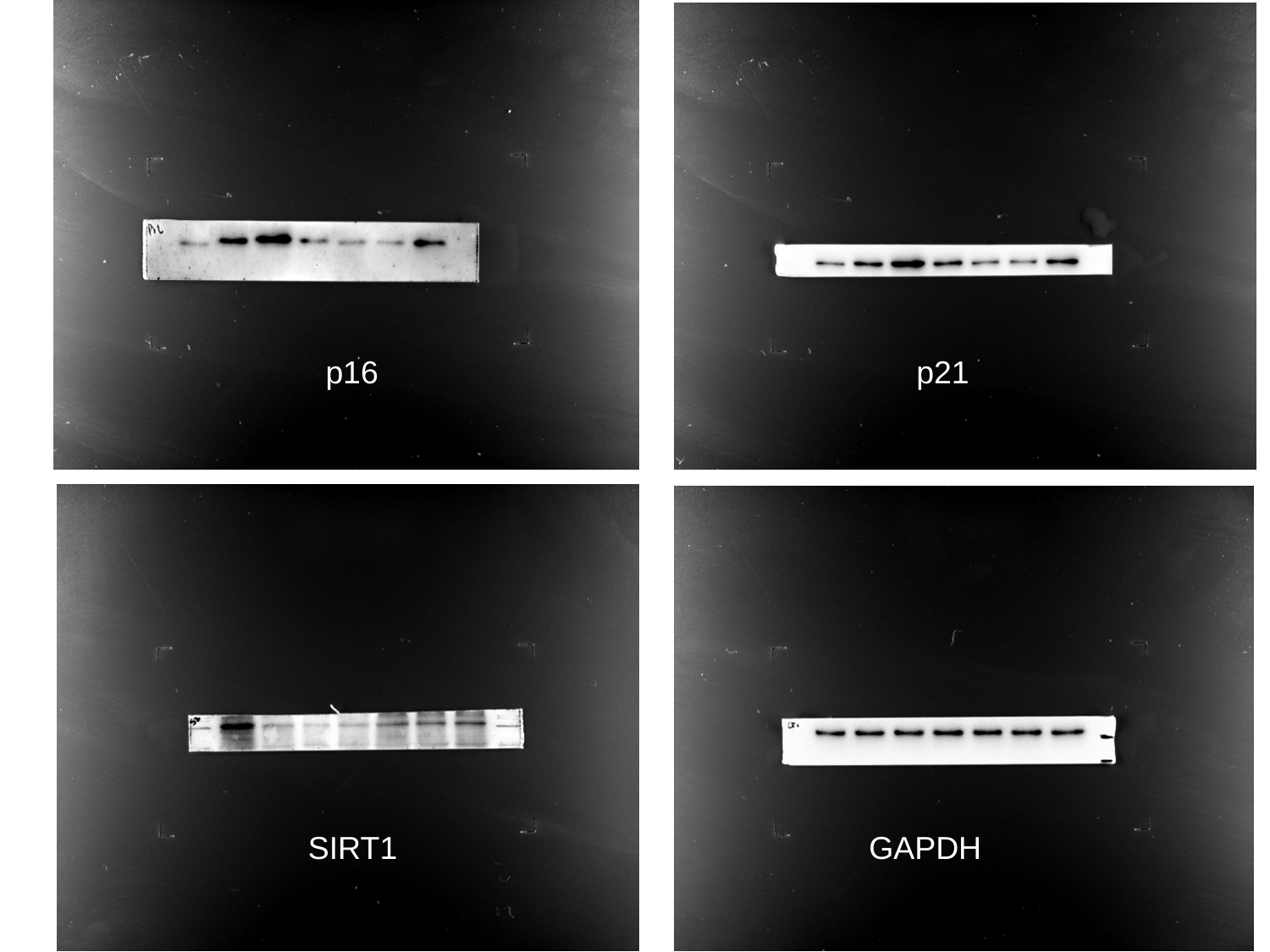

#
p16
p21
SIRT1
GAPDH

## Slide 2
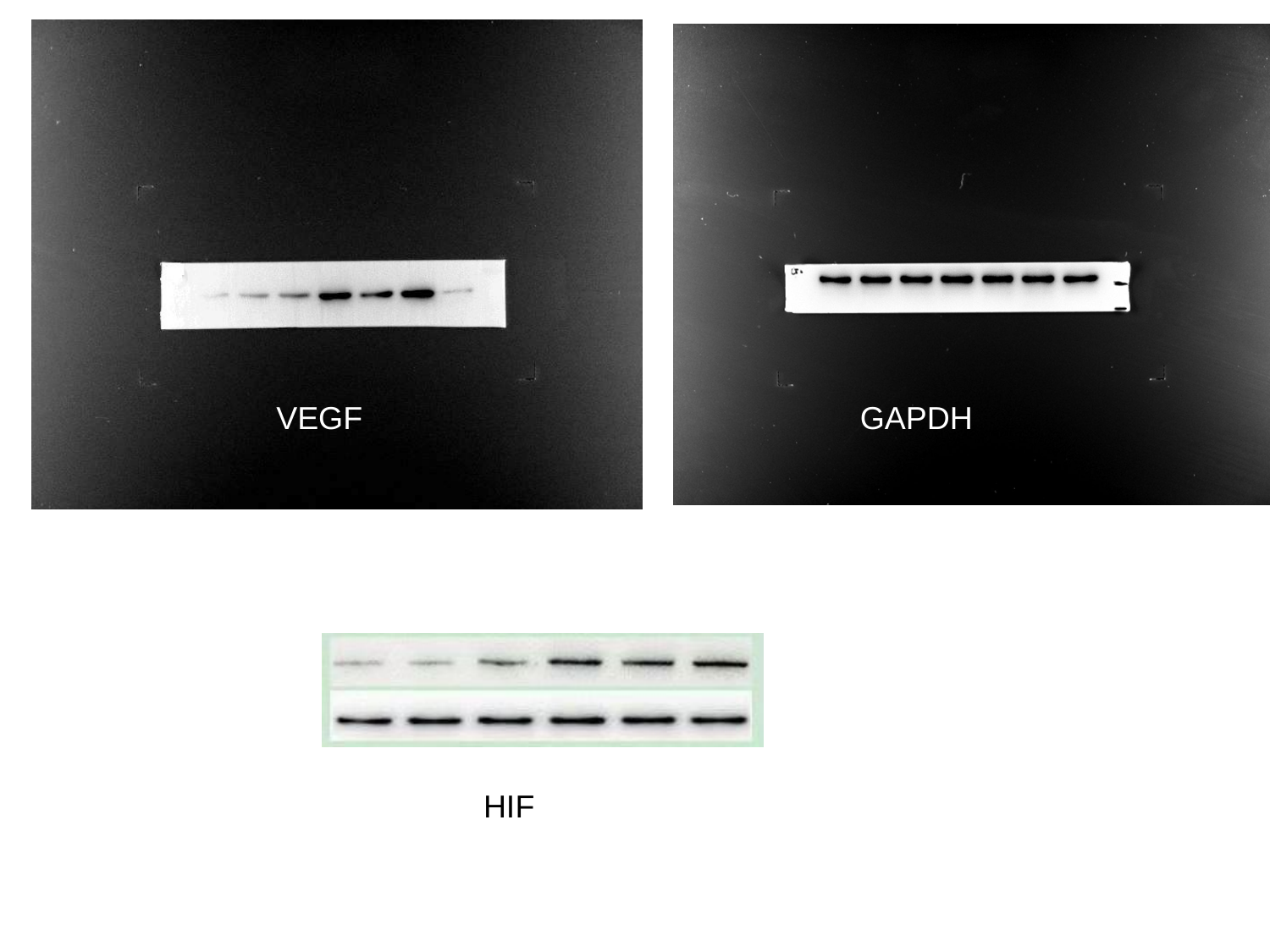

#
VEGF
GAPDH
HIF

Supplement: Supplementary file 1 [file presentation1.ppt]
